# Relação entre problemas de sono e padrão de atividade física de universitários na pandemia

**DOI:** 10.15446/rsap.V25n4.102071

**Published:** 2023-07-01

**Authors:** Luciano Fiorentin, Sirlei Favero Cetolin, Luana Patrícia Marmitt, Vilma Beltrame

**Affiliations:** 1 LF: Enf. M.Sc. Biociências e Saúde. Universidade do Oeste de Santa Catarina, Joaçaba-SC, Brasil. fiorentinl@yahoo.com.br Universidade do Oeste de Santa Catarina Universidade do Oeste de Santa Catarina Joaçaba SC Brasil; 2 SF: Assistente Social. Psc. Docente, Programa de Mestrado Biociências e Saúde, Universidade do Oeste de Santa Catarina. Joaçaba - SC, Brasil. sirleicetolin@gmail.com Universidade do Oeste de Santa Catarina Programa de Mestrado Biociências e Saúde Universidade do Oeste de Santa Catarina Joaçaba SC Brasil; 3 LM: Nutr. Docente, Programa de Mestrado Biociências e Saúde. Universidade do Oeste de Santa Catarina, Joaçaba-SC, Brasil. luana.marmitt@unoesc.edu.br Universidade do Oeste de Santa Catarina Programa de Mestrado Biociências e Saúde Universidade do Oeste de Santa Catarina Joaçaba SC Brasil luana.marmitt@unoesc.edu.br; 4 VB: Enf. Docente, Programa de Mestrado Biociências e Saúde, Universidade do Oeste de Santa Catarina. Joaçaba-SC, Brasil. vilma.beltrame@unoesc.edu.br Universidade do Oeste de Santa Catarina Programa de Mestrado Biociências e Saúde Universidade do Oeste de Santa Catarina Joaçaba SC Brasil vilma.beltrame@unoesc.edu.br

**Keywords:** Exercício físico, comportamento sedentário, estudantes de ciências da saúde *(fonte: DeCS, BIREME)*, Exercise, sedentary behavior, students, health occupations *(source: MeSH, NLM)*, Ejercicio físico, conducta sedentaria, estudiantes del área de la salud *(fuente: DeCS, BIREME)*

## Abstract

**Objetivo:**

Objetivou-se caracterizar universitários da saúde que na pandemia da Covid-19 adquiriram problemas de sono e aqueles inativos fisicamente, relacionando essas condições.

**Métodos:**

Os dados foram coletados em 2020 por meio eletrônico. Para caracterização dos problemas de sono considerou-se: dificuldades para iniciar, insatisfação com o sono e se manter dormindo, se o tempo de sono é suficiente para se sentir bem e problemas de sono adquiridos durante a pandemia. Para a inatividade física o Questionário Internacional de Atividade Física. Usou-se o teste de qui-quadrado de heterogeneidade e de tendência linear.

**Resultados:**

Participaram 656 estudantes. A prevalência de problemas de sono foi de 48,8%, e inatividade física 54,6%. Ambas mais frequentes nos mais velhos e que relataram piora da alimentação (p<0,05). O maior tempo de distanciamento social foi associado à inatividade física (p=0,020), enquanto maior sensação de ansiedade, tristeza ou preocupação (p=0,013) e maior consumo de álcool aumentaram os problemas de sono (0,031).

**Conclusões:**

Conclui-se que problemas do sono e inatividade física foram mais freuentes em estudantes mais velhos e com piora alimentar. O maior tempo de distanciamento social foi associado à inatividade física, e o aumento da ansiedade, tristeza ou preocupação e do consumo de álcool aumentaram os problemas de sono.

## Abstract (en)

**Objective:**

The objective of this study was to characterize health students who in the Covid-19 pandemic acquired sleep problems and those physically inactive, relating these conditions.

**Methods:**

Data were collected in 2020 electronic. To characterize sleep problems, we considered: difficulties to start, dissatisfaction with sleep and staying asleep, whether the sleep time is enough to feel good and sleep problems acquired during the pandemic. For physical inactivity, the International Physical Activity Questionnaire. The chi-square test of heterogeneity and linear trend was used.

**Results:**

Participated 656 students. The prevalence of sleep problems was 48.8%, and physical inactivity 54.6%. Both more frequent in older patients who reported worsening of feeding (p<0.05). Longer social distance was associated with physical inactivity (p=0.020), while greater feeling of anxiety, sadness or worry (p=0.013) and higher alcohol consumption increased sleep problems (0.031).

**Conclusions:**

It was concluded that sleep problems and physical inactivity were more frequent in older students with worsening diet. Longer social distance was associated with physical inactivity, and increased anxiety, sadness or worry and alcohol consumption increased sleep problems.

## RESUMEN

**Objetivo:**

El objetivo de este estudio fue caracterizar a los estudiantes de salud que adquirieron problemas de sueño durante la pandemia de covid-19, como también a los físicamente inactivos, relacionando estas condiciones.

**Métodos:**

Los datos fueron recolectados electrónicamente en el 2020. Para caracterizar los problemas de sueño, se consideraron: dificultades para comenzar, insatisfacción con el sueño y permanecer dormido, si el tiempo de sueño es suficiente para sentirse bien y problemas de sueño adquiridos durante la pandemia. Para la inactividad física, el Cuestionario Internacional de Actividad Física. Se utilizó la prueba chi-cuadrado de heterogeneidad y tendencia lineal.

**Resultados:**

Participaron 656 estudiantes. La prevalencia de problemas de sueño fue del 48,8 % y la inactividad física del 54,6 °%, ambos más frecuentes en pacientes mayores, los cuales reportaron empeoramiento de la alimentación (p < 0,05). Una mayor distancia social se asoció con inactividad física (p=0,020), mientras que una mayor sensación de ansiedad, tristeza o preocupación (p=0,013) y un mayor consumo de alcohol aumentaron los problemas de sueño (0,031).

**Conclusiones:**

Se concluyó que los problemas de sueño y la inactividad física eran más frecuentes en los estudiantes mayores con empeoramiento de la dieta. Una mayor distancia social se asoció con la inactividad física, y el aumento de la ansiedad, la tristeza o la preocupación y el consumo de alcohol aumentaron los problemas de sueño.

Aprática de atividade física na vida dos estudantes tem relevância especial, pois além dos benefícios à saúde, como a manutenção do sistema cardiovascular, contribui para as funções cognitivas 1). Além da atividade física, também, a qualidade do sono está relacionada ao equilíbrio das funções fisiológicas e ao desempenho do aprendizado 2.

Em estudo de meta-análise de relações entre atividade física e qualidade do sono em estudantes universitários constatou-se que a atividade física de intensidade vigorosa e moderada foram associadas à melhor qualidade do sono 3. O inverso também se confirmou, em estudo com estudantes universitários na Arábia Saudita, onde a boa qualidade do sono foi associada ao maior número de dias de atividade física 4.

A presença de elevados índices de problemas de sono, já tem sido evidenciada em estudos desenvolvidos com estudantes de ciências de saúde 5,6. Não diferente, achados demonstram a presença de taxas consideráveis também de inatividade física presentes na rotina de universitários 7.

Durante o período de restrições em virtude da pandemia pela Covid-19, foram suspensas as atividades não essenciais como aulas em escolas e universidades, bem como academias 8. Tal quadro reduziu ainda mais as práticas de atividade física dos universitários e da população geral 9. Estimativas indicam que a inatividade física teve acréscimo de 26% na população adulta brasileira 10. Da mesma forma, devido às preocupações em relação à infecção pelo novo Coronavírus, o isolamento social e a ansiedade, os problemas relacionados ao sono também foram agravados 11.

Assim, diante das evidências já expostas, os estudantes universitários tiveram a condição do sono e atividade física agravadas pelos reflexos da pandemia da covid-19. Dessa forma, torna-se relevante a identificação dos grupos que apresentaram maiores problemas com o sono e inatividade física durante a pandemia. Com essa identificação, torna-se possível estruturar intervenções para esses grupos, permitindo condições adequadas de bem-estar e qualidade de vida aos estudantes.

Dessa forma, o objetivo desse estudo foi caracterizar os estudantes universitários dos cursos de ciências da saúde que durante a pandemia da Covid-19 adquiriram problemas de sono e aqueles inativos fisicamente, relacionando ambas as condições.

## METODO

Estudo epidemiológico transversal, resultante de um recorte de dissertação de mestrado, realizado no período de julho a setembro de 2020, com alunos de 18 anos ou mais matriculados nos cursos de graduação e pós-graduação da área da saúde da Universidade do Oeste de Santa Catarina (UNOESC) e da Universidade Católica de Santa Catarina. Ambas as universidades representam instituições de ensino comunitárias localizadas no meiooeste do estado de Santa Catarina, Brasil e tiveram as aulas presenciais suspensas durante o período em que os dados foram coletados em virtude das medidas de distanciamento social pela Covid-19.

O tamanho amostral foi calculado a partir do total de 4 700 estudantes que representavam o total da população-alvo nos locais de estudo. Com base no nível de confiança de 95 % e com distribuição heterogênea, o número mínimo de participantes necessário para a pesquisa foi de 571 universitários.

O instrumento de coleta de dados foi estruturado em três partes. A primeira, refere-se aos dados sobre a idade, sexo, universidade, curso, trabalho e tempo de distanciamento social. Também foram coletadas informações a respeito de comportamentos de antes e durante a pandemia, como percepção da qualidade alimentar, consumo de álcool, e presença de ansiedade, tristeza e preocupação. Na segunda parte, coletou-se dados sobre o sono (dificuldades para iniciar o sono e se manter dormindo, insatisfação com o sono atual, tempo em horas de sono, se o tempo de sono é suficiente para se sentir bem, e problemas com o sono adquirido durante a pandemia - esse sendo o primeiro desfecho deste estudo). Na terceira parte, o padrão de atividade física foi investigado a partir do questionário IPAQ Short - *International Physical Activity Questionnaire Short*^12^, o qual considera: ativo aquele que praticou no mínimo 150 minutos de atividade física em pelo menos cinco dias da semana; Insuficientemente ativos os que não atingiram o mínimo descrito anteriormente; e inativos os que não realizam atividade física em nenhuma quantidade (que constituiu o segundo desfecho deste estudo).

O questionário foi enviado a todos os estudantes dos cursos das ciências da saúde de ambas as universidades de forma online pela plataforma do Google forms, juntamente com o termo de Consentimento Livre e Esclarecido.

Os dados da plataforma online foram transferidos para o programa Stata 13.0 onde seguiram-se as análises estatísticas. Essas constataram descrição da amostra, onde utilizou-se números absolutos e proporções. Para as análises de associação de cada desfecho (problemas de sono e inatividade física) e variáveis independentes utilizou-se o teste de qui-quadrado de heterogeneidade e de tendência linear. Ambos os desfechos foram ainda relacionados por meio do mesmo teste de heterogeneidade e ilustrados em gráficos de barras estratificados pelo sexo do estudante. O nível de significância estatística adotado foi de 5%.

O projeto de pesquisa foi aprovado pelo Comitê de Ética em Pesquisa da Unoesc/Hust, com parecer N.° 4067646.

## RESULTADOS

Retornaram 723 questionários, foram excluídos 67 que não representavam cursos da saúde, respostas incompletas e possuírem idade inferior a 18 anos, totalizando 656 participantes. A maioria era do sexo feminino (84,8%), com idade média de 23,8 (±6,6) anos. Dentre os cursos, 31,5 % (n=207) dos estudantes cursavam psicologia, 13,4% (n=88) medicina e 11,1 % (n=73) enfermagem. Além disso, 47,9% (n=314) trabalhavam concomitante ao estudo. Durante a pandemia, 67,2% (n = 441) dos estudantes permaneceram mais de dois meses em distanciamento social e 47,9% (n = 314) saíam de casa somente para atividades essenciais. Nesse período, 43,9 % (n=288) permaneceram mais de 5 horas diárias conectados às mídias sociais e, 18,7% mais de 7I1 diárias. Aumento na ansiedade, tristeza ou preocupação foi constatado por 83,7% (n=559), piora da qualidade alimentar por 37,4% (n=245), e maior consumo de álcool por 13,9% (n=91). Problemas com o sono esteve presente em 48,8 % dos estudantes, sendo 41 % (n=131) destes adquiridos na pandemia. A inatividade física foi observada em 54,6% (n=358) dos estudantes e 32,2 % (n=211) foram insuficientemente ativos. (Tabela 1).

**Tabela 1:** Descrição da amostra de estudantes universitários participantes do estudo. Joaçaba, SC, 2020. (n = 656)

Variável	n	%
Sexo		
Feminino	556	84,8
Masculino	100	15,2
Idade (anos completos)		
18 a 19	159	24,2
20 a 21	178	27,1
22 a 23	119	18,1
24 ou mais	200	30,5
Curso de graduação ou pós-graduação		
Psicologia	207	31,6
Medicina	88	13,4
Enfermagem	73	11,1
Nutrição	41	6,3
Educação Física	39	5,9
Odontologia	39	5,9
Outros	169	25,8
Trabalha	314	47,9
Distanciamento social		
Sim. Saiu de casa apenas para atividades essenciais	314	47,9
Sim, parcialmente. Deixou de fazer algumas coisas	212	32,3
Sim, mas logo voltou às atividades normais	111	16,9
Não, em nenhum momento	19	2,9
Tempo de distanciamento		
Até 3 semanas	139	21,1
1 mês	76	11,6
2 meses ou mais	441	67,2
Tempo diário conectado às mídias sociais		
Até 2 horas	110	16,8
3 a 4 horas	258	39,3
5 a 6 horas	165	25,2
7 horas ou mais	123	18,7
Maior sentimento de ansiedade, tristeza ou preocupação em comparação a antes da pandemia		
Percepção da qualidade da alimentação em comparação a antes da pandemia	549	83,7
Tem se alimentado igual	292	44,5
Passou a se alimentar melhor	119	18,1
Passou a se alimentar pior	245	37,4
Consumo de bebidas alcoólicas em comparação a antes da pandemia		
Não consome álcool	218	33,2
Aumentou o consumo de álcool	91	13,9
Reduziu o consumo de álcool	137	20,9
Consumo igual	210	32,0
Problemas relacionados ao sono		
Não tem	336	51,2
Sim, já tinha	189	28,8
Sim, adquiriu na pandemia	131	20,0
Padrão de atividade física (IPAQ)		
Ativos fisicamente - pelo menos 30 minutos por dia de atividade física moderada durante cinco ou mais dias por semana	87	13,3
Insuficientemente ativos - não alcançam a recomendação	211	32,2
Inativos - não praticam nenhuma quantidade de atividade física	358	54,6
Total	656	100,0

A inatividade física no período de distanciamento social foi significativamente associada á idade, sendo mais comum nos estudantes com 24 anos ou mais (p=0,038), além de maior tempo de distanciamento social (até três semanas) (p=0,020), e percepção de piora na qualidade alimentar durante a pandemia (p<0,001). Já os problemas relacionados ao sono adquiridos durante a pandemia foram também associados à idade (p = 0,005), com maior frequência entre estudantes de 22 a 23 anos (26,1%), além do aumento dos sentimentos de ansiedade, tristeza ou preocupação (p=0,013), redução da qualidade alimentar (p=0,017) e aumento no consumo de álcool (p=0,031) (Tabela 2).

**Tabela 2:** Características dos estudantes de acordo com os problemas de sono adquiridos na pandemia e a inatividade física. Joaçaba, SC, 2020 (n=656)

Variável	Inatividade física (n = 358)		Problemas de sono adquiridos (n = 131)	
	(%)	Valor p*	(%)	Valor p*
Sexo		0,099		0,420
Feminino	55,9		20,5	
Masculino	47,0		17,0	
Idade (anos completos)		0,038		0,005
18 a 19	50,3		20,7	
20 a 21	48,3		11,2	
22 a 23	58,0		26,1	
24 ou mais	61,5		23,5	
Trabalho		0,920		0,190
Não	54,4		21,9	
Sim	54,8		17,8	
Distanciamento social		0,450		0,236
Sim. Saiu de casa apenas para atividades essenciais	68,4		31,6	
Sim, parcialmente. Deixou de fazer algumas coisas	50,4		20,7	
Sim, mas logo voltou às atividades normais	53,3		16,0	
Não, em nenhum momento	56,1		21,7	
Tempo de distanciamento		0,020#		0,453
Até 3 semanas	63,2		23,7	
1 mês	55,3		18,4	
2 meses ou mais	51,9		19,1	
Tempo diário conectado às mídias sociais		0,869		0,192
Até 2 horas	57,3		15,4	
3 a 4 horas	53,9		21,3	
5 a 6 horas	52,7		17,0	
7 horas ou mais	56,1		25,2	
Maior sentimento de ansiedade, tristeza ou preocupação em comparação a antes da pandemia		0,117		0,013
Não	47,7		11.2	
Sim	55,9		21,7	
Percepção da qualidade da alimentação em comparação a antes da pandemia		< 0,001		0,017
Tem se alimentado igual	56,5		15,4	
Passou a se alimentar melhor	38,7		20,2	
Passou a se alimentar pior	60,0		25,3	
Consumo de bebidas alcoólicas em comparação a antes da pandemia		0,187		0,031
Não consome álcool	52,3		21,6	
Consumo igual	57,6		13,8	
Aumentou o consumo de álcool	61,5		27,5	
Reduziu o consumo de álcool	48,9		21,9	

*Qui-quadrado de heterogeneidade; #Tendência linear.

**Figura 1:**
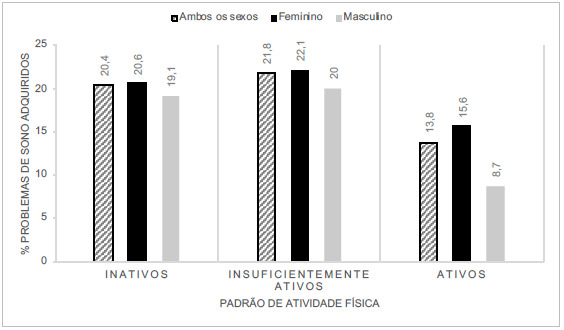
Relação entre os problemas relacionados ao sono adquiridos na pandemia e padrão de atividade física na amostra estratificado por sexo. Joaçaba, SC, 2020 (n=656)

A Figura 1 que relaciona ambos os desfechos, demonstra maior prevalência de problemas de sono adquiridos durante a pandemia entre os estudantes com padrão de atividade física insuficientemente ativos (21,8 %>) e inativos (20,4 %>). Cerca de 20 %> dos estudantes tinham ambos os desfechos, ou sejam, eram inativos e desenvolveram problemas de sono na pandemia (20,4 %). Na análise estratificada, o sexo feminino desenvolveu mais problemas do sono na pandemia quando comparado ao masculino, independentemente da classificação do padrão de atividade física.

## DISCUSSÃO

O sono de qualidade e a prática de atividade física atuam de forma independente no restabelecimento, manutenção e proteção de diversos aspectos dos sistemas corporais, associados a diversos aspectos do bem-estar e qualidade de vida. Entre suas funções, estão o revigoramento e capacidade de regulação da homeostase e plasticidade cerebral, fundamentais para organização cognitiva, melhora no aprendizado e prevenção de diversas doenças crônicas, 2,13 reforçando a relevância do sono e da atividade física na rotina dos estudantes.

Apesar dos benefícios, estudos têm demonstrado índices elevados de qualidade do sono ruim e inatividade física entre os universitários, condições que os expõem ao risco de comprometimento da saúde e bem-estar físico e psicológico 6,14.

Os resultados obtidos neste estudo retratam os problemas do sono e da inatividade física durante o período de pandemia por Covid-19, em que os estudantes estavam condicionados às restrições do distanciamento social, normatizado por portaria do estado de Santa Catarina 15, piorando a condição do sono e os padrões de atividade física insuficientemente ativo e inativo em relação a antes da pandemia. Resultado semelhante foi evidenciado também em universitários do Canadá 16, mas o oposto foi observado em estudantes de ciências da saúde da Espanha, onde o tempo de distanciamento social elevou o tempo gasto com atividade física, embora o tempo sentado também tenha aumentado simultaneamente 11.

A inatividade física foi prevalente em 54,6% dos estudantes. Estudo realizado com universitários no Canadá para comparar atividade física e sedentarismo antes e durante a pandemia demonstrou que anteriormente à pandemia, 16% eram ativos, reduzindo para 9,6%, como consequência, elevando o comportamento sedentário 16. Esses resultados possivelmente refletem o fechamento de academias, restrição de circulação em praças e parques e ao confinamento realizado pelos estudantes. De fato, quase metade da amostra (47,9%) relatou sair de casa apenas para atividades essenciais, o que dificulta a prática de atividade física.

Em relação aos problemas relacionados ao sono, 20 % referiram tê-los desenvolvido no período da pandemia. O aumento de problemas relacionados ao sono foi encontrado em estudo com estudantes de enfermagem realizado na Espanha. Com registros de duas coletas de dados, antes e durante o distanciamento social, a qualidade do sono ruim subiu 7,1 % em relação à antes da pandemia, 11 confirmando os efeitos negativos da pandemia no sono dos universitários. Estes problemas tendem a aumentar, pois a queda na qualidade alimentar, aumento do consumo de álcool, aumento dos sentimentos de ansiedade, tristeza ou preocupação, foram originados ou agravados durante a pandemia e demonstraram ser fator de risco ao desenvolvimento dos problemas com o sono.

Já os problemas relacionados ao sono adquiridos durante a pandemia foram também associados à idade (p=0,005), cuja maior frequência entre estudantes de 22 a 23 anos (26,1%), além do aumento dos sentimentos de ansiedade, tristeza ou preocupação (p=0,013), redução da qualidade alimentar (p=0,017) e aumento no consumo de álcool (p=0,031)

A idade e a qualidade da alimentação foram associadas tanto à inatividade física quanto à aquisição de problemas de sono. Conforme o aumento da idade, maior a prevalência encontrada de inatividade física, semelhante ao resultado encontrado no estudo de Gallè et al. 17, onde ter menos de 22 anos foi associado a alcançar níveis satisfatórios de atividade física. Já nos problemas com o sono, houve oscilações entre faixas etárias, e a maior prevalência esteve nos estudantes de 22 e 23 anos.

Entre os estudantes que se alimentaram pior na pandemia, a inatividade física e aquisição de problemas com o sono foi mais frequente. Estudo transversal realizado com universitários e com abrangência multicêntrica durante a pandemia, identificou que estudantes que apresentaram piora na qualidade do sono, tiveram maiores escores de riscos alimentares 18. Estudo de Ogilvie; Patel 19, sugerem a relação de sono insatisfatório com alimentação de baixa qualidade e ganho de peso. Du et al. 20, consideraram a qualidade do sono um mediador entre comportamentos de estresse e os comportamentos alimentares. Ainda, resultado de estudo com estudantes de enfermagem da Universidade de Castilla-La Mancha (Espanha) que investigou associações à dependência alimentar, encontraram relações com sedentarismo, e sugerem que a dependência alimentar é consequência de estilos de vida e comportamentos pouco saudáveis 21, como é o caso da inatividade física identificada neste estudo.

O maior tempo de distanciamento social foi associado à inatividade física entre os estudantes. Estudo que envolveu 1430 universitários italianos demonstrou que dentre as associações à redução da atividade física na pandemia se deu entre estudantes que costumavam desenvolver suas práticas com amigos, grupos sociais ou exercícios de envolvimento coletivos e em ambientes esportivos 17, o qual, em respeito às restrições sanitárias devido à Covid-19, deixaram de acontecer.

No período em que permaneceram sobre as restrições do distanciamento social, observou-se uma elevada prevalência (83,7%) de aumento do sentimento de ansiedade, tristeza ou preocupação comparado à antes da pandemia. Essa condição foi relacionada aos problemas com o sono adquiridos na pandemia pelos estudantes deste estudo. Os resultados do estudo transversal de base populacional realizado em Campinas (SP) que avaliou qualidade do sono, saúde e bem-estar, associou o sono de qualidade ruim à situação que resultam na insatisfação com a vida 22.

Observou-se associação entre o consumo de álcool e os problemas de sono adquiridos na pandemia. Esse resultado foi corroborado por pesquisa realizada com estudantes da enfermagem na pandemia 11, onde a maior prevalência de qualidade ruim do sono se deu entre aqueles que aumentaram o consumo de álcool. O excesso de álcool provoca alterações na arquitetura do sono. O tempo de início do sono é diminuído, mas não consegue manter-se dormindo. Ou seja, melhora para iniciar o sono, mas acorda antes do final da noite 23, fato que pode explicar a insatisfação com o sono relatado por alguns estudantes deste estudo. O uso de álcool não apresentou associação com a inatividade física, mas em estudo anterior o uso de álcool foi associado ao aumento de permanência de tempo sentado 24.

A atividade física melhora a percepção da qualidade do sono 25, entretanto, essa relação está associada a níveis de atividade física ativo 26 e intensidade moderada a vigorosas 3,26, assim como o sono de boa qualidade está associado ao maior número de dias de práticas de atividade física 4. Exercícios aeróbicos regulares e com aumento de intensidade possuem recomendação para manutenção da qualidade do sono 27.

No nosso estudo, os problemas relacionados ao sono adquiridos na pandemia estão presentes em todos os padrões de atividade física. Apesar de não haver uma explicação clara, os resultados mostraram que os estudantes com padrão de atividade física ativos foram os que apresentaram os menores níveis de prevalência aos problemas com o sono neste período. Resultados de estudo com 2100 universitários croatas mostraram que a qualidade do sono ruim está associada a atividade física insuficiente 28.

Os problemas do sono adquiridos durante a pandemia e a inatividade física, ainda que de forma independente, interferem na qualidade de vida dos estudantes de ciências da saúde. Há ainda que se considerar que um quinto dos estudantes apresentaram ambas as condições, inatividade física e problemas de sono de forma concomitante. As relações bidirecionais de associações ou de causalidade ainda precisam ser elucidadas.

### Limitações

Sendo um estudo de delineamento transversal, sua principal limitação é a impossibilidade de estabelecer a relação causal entre os desfechos, e pelo fato de as respostas serem subjetivas, não se descarta possíveis vieses de confusão interpretativas nas respostas entre os pesquisados.

Foram identificadas altas prevalências de problemas de sono e inatividade física tanto de forma independente, quanto à apresentação de ambas as condições, que atingiram cerca de 20 % da amostra. Ambas os desfechos foram significativamente mais frequentes entre estudantes mais velhos, que tiveram piora da qualidade da alimentação na pandemia. O maior tempo de distanciamento social foi associado somente à inatividade física, enquanto maior sensação de ansiedade, tristeza ou preocupação e maior consumo de álcool aumentaram significativamente os problemas de sono.

Os problemas do sono adquiridos na pandemia estão presentes em todos os padrões de atividade física, sendo mais prevalentes nos estudantes insuficientemente ativos e inativos de ambos os sexos.

Estratégias de intervenção deverão focar os grupos de estudantes mais velhos, que apresentam tendências à irregularidade de hábitos alimentares, praticam pouco ou não praticam atividade física, apresentam facilidade de aumento de sentimento de ansiedade, tristezas e consumo abusivo de álcool.

Recomenda-se assim, que as universidades elaborem planos transversais, de abordagem multidisciplinar, para promover a qualidade do sono, estimular as práticas de atividade física, em todos os eventos acadêmicos, independente da área de abrangência. Também, através do centro de apoio ao estudante, desenvolver programas educativos e terapêuticos, focados nos estudantes pertencentes aos grupos que apresentaram maior risco de desenvolver problemas com o sono e inatividade física ♦
